# Concomitant targeting of the mTOR/MAPK pathways: novel therapeutic strategy in subsets of *RICTOR/KRAS*-altered non-small cell lung cancer

**DOI:** 10.18632/oncotarget.26129

**Published:** 2018-09-21

**Authors:** Dennis Ruder, Vassiliki Papadimitrakopoulou, Kazuhiko Shien, Carmen Behrens, Neda Kalhor, Huiqin Chen, Li Shen, J. Jack Lee, Waun Ki Hong, Ximing Tang, Luc Girard, John D. Minna, Lixia Diao, Jing Wang, Barbara Mino, Pamela Villalobos, Jaime Rodriguez-Canales, Nana E. Hanson, James Sun, Vincent Miller, Joel Greenbowe, Garrett Frampton, Roy S. Herbst, Veera Baladandayuthapani, Ignacio I. Wistuba, Julie G. Izzo

**Affiliations:** ^1^ Graduate Program in Human and Molecular Genetics and Cancer Biology, MD Anderson Cancer Center UTHealth Graduate School of Biomedical Sciences, Houston, Texas, USA; ^2^ Department of Translational Molecular Pathology, The University of Texas MD Anderson Cancer Center, Houston, Texas, USA; ^3^ Department of Bioinformatics and Computational Biology, The University of Texas MD Anderson Cancer Center, Houston, Texas, USA; ^4^ Department of Thoracic/Head and Neck Medical Oncology, The University of Texas MD Anderson Cancer Center, Houston, Texas, USA; ^5^ Department of Pathology, The University of Texas MD Anderson Cancer Center, Houston, Texas, USA; ^6^ Department of Biostatistics, The University of Texas MD Anderson Cancer Center, Houston, Texas, USA; ^7^ Hamon Center for Therapeutic Oncology Research, The University of Texas Southwestern Medical Center, Dallas, Texas, USA; ^8^ Yale Cancer Center, Yale School of Medicine, New Haven, Connecticut, USA; ^9^ Foundation Medicine, Inc., Cambridge, Massachusetts, USA

**Keywords:** RICTOR gene abnormalities, KRAS mutation, MAPK pathway, mTORC2, non-small cell lung cancer

## Abstract

Despite a therapeutic paradigm shift into targeted-driven medicinal approaches, resistance to therapy remains a hallmark of lung cancer, driven by biological and molecular diversity. Using genomic and expression data from advanced non-small cell lung cancer (NSCLC) patients enrolled in the BATTLE-2 clinical trial, we identified *RICTOR* alterations in a subset of lung adenocarcinomas and found *RICTOR* expression to carry worse overall survival. RICTOR-altered cohort was significantly enriched in *KRAS/MAPK* axis mutations, suggesting a co-oncogenic driver role in these molecular settings. Using NSCLC cell lines, we showed that, distinctly in *KRAS* mutant backgrounds, *RICTOR* blockade impairs malignant properties and generates a compensatory enhanced activation of the MAPK pathway, exposing a unique therapeutic vulnerability. *In vitro* and *in vivo* concomitant pharmacologic inhibition of mTORC1/2 and MEK1/2 resulted in synergistic responses of anti-tumor effects. Our study provides evidence of a distinctive therapeutic opportunity in a subset of NSCLC carrying concomitant *RICTOR/KRAS* alterations.

## INTRODUCTION

Despite improvement in early detection strategies and standard treatment options, NSCLC continues to have a poor prognosis [1, 2]. Once considered a single disease entity, NSCLC is comprised of discrete genetic, biologically functional, and clinically distinct subgroups [3]. With increased knowledge of genomic aberrations, the last decade enabled development of rapid genomic profiling leading to molecular-targeted therapies blocking key oncogenic drivers and resulting in dramatic responses in selected patients [2]. Despite initial responses, these targeting agents rarely promote complete or durable anti-tumor effects especially in unselected patients, leading to acquired resistance mechanisms and relapse. Further, effective therapeutic options are still lacking for lung tumors driven by other key mutations such as oncogenic *KRAS* (~30%) and those with unknown oncogenic drivers [4, 5].

To identify novel actionable targets, we queried targeted next-generation sequencing and gene expression profiling data associated with the BATTLE-2 trial (BATTLE-2: A Biomarker-Integrated Targeted Therapy Study in Previously Treated Patients With Advanced Non-Small Cell Lung Cancer), an umbrella study of targeted therapy focusing on *KRAS* mutated (mut*KRAS*) cancers [6]. Our approach was two-fold. First, we focused solely on the adenocarcinoma (LUAD) subtype, the majority of the enrolled cases. Second, we searched for genes harboring both gene amplifications and somatic mutations, a genetic pattern recognized as a hallmark of potential driver oncogenes or co-oncogenes. We identified a subgroup (17%) of patients with genomic alterations of *RICTOR* (rapamycin-insensitive companion of mTOR), a main structural/functional subunit of mTORC2 (mammalian target of rapamycin, complex 2) and critical node of the PI3K/AKT/mTOR pathway [7, 8]. Collectively, mTORC2 targets are involved in cell survival, proliferation, stress response and actin-cytoskeletal reorganization [9–12]. RICTOR's oncogenic role has gained momentum over recent years, with reports uncovering both canonical rate limiting activity on mTORC2-AKT, and the presence of additional mTORC2-independent functionalities [13–16].

We aimed to understand the clinical significance of *RICTOR* alterations, and to define settings where RICTOR or RICTOR-associated signaling blockade might enhance conventional targeted therapy approaches in LUAD. We surveyed clinical-molecular databases associated with advanced stage (BATTLE-2), surgically resected (PROSPECT) and early stage (The Cancer Genome Atlas = TCGA) LUAD cases. Subsequently, we used a panel of *RICTOR* amplified and non-amplified NSCLC cells to characterize phenotypic and molecular consequences of RICTOR blockade *in vitro* and *in vivo*. Further, we investigated the survival compensatory pathways associated with RICTOR blockade, and we took a dual pathway inhibition approach to blunt rescue mechanism(s) to produce significant anti-tumor effects *in vitro* and *in vivo*.

## RESULTS

### RICTOR genomic alterations are present in early and advanced lung adenocarcinoma, but RICTOR expression portends worse outcome in late stage

**Figure 1:**
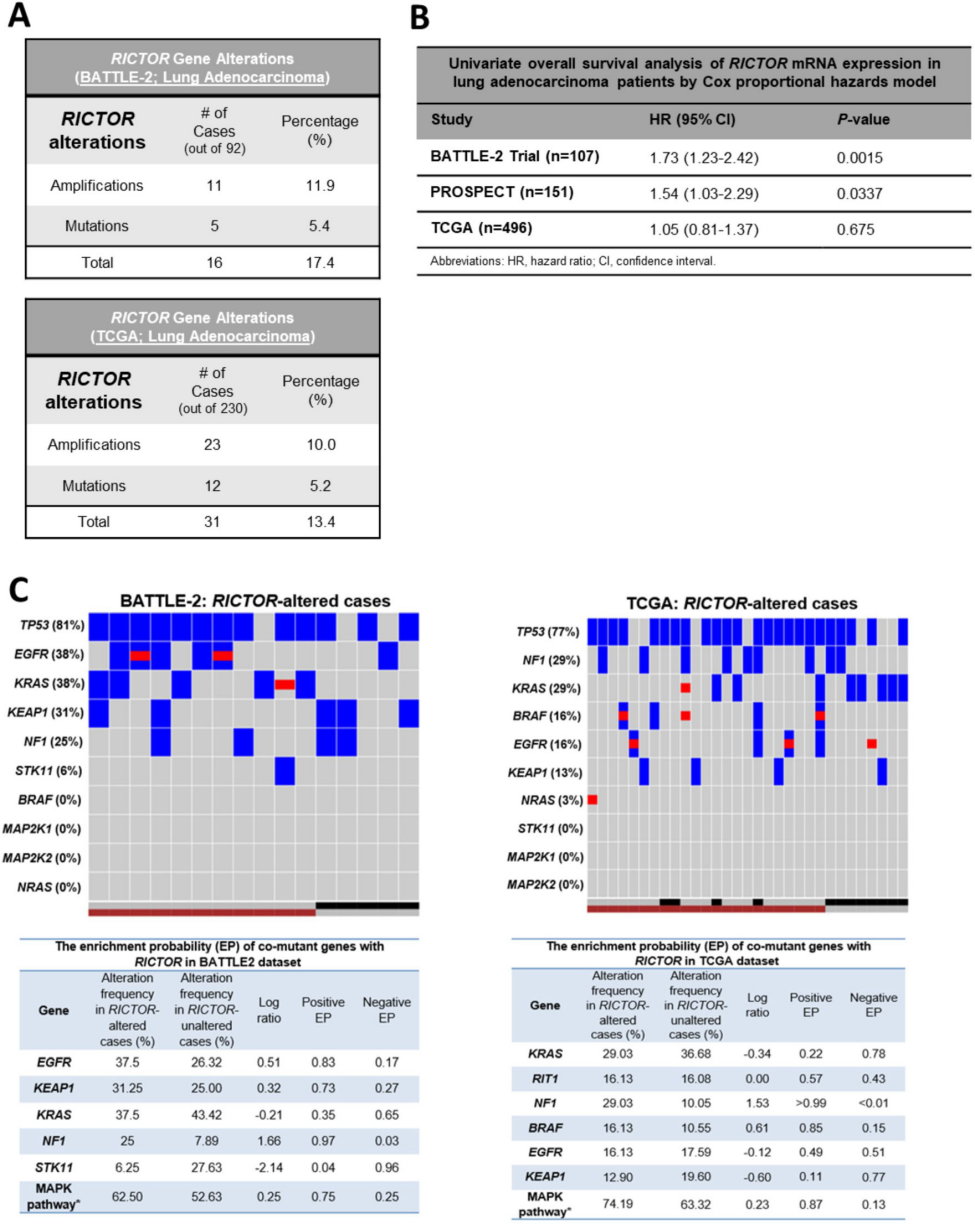
*RICTOR* alterations are present in early and advanced stage lung adenocarcinomas and are co-enriched with MAPK pathway alterations (**A**) Summary of frequency of *RICTOR* gene alterations (mutations or amplifications) in chemo-refractory advanced lung adenocarcinoma samples (BATTLE-2, *n* = 92) and in surgically resected lung adenocarcinoma samples (TCGA, *n* = 230). (**B**) Univariate Cox proportional hazards regression analysis of *RICTOR* mRNA expression in lung adenocarcinoma cases from the BATTLE-2, PROSPECT, and TCGA datasets. HR, hazard ratio; CI, confidence interval. (**C**) Co-mutational landscape in *RICTOR* genomic altered cases: Top Panels – Heat Maps for BATTLE-2 and TCGA LUAD cases. *RICTOR* amplification = maroon square; *RICTOR* mutant = black square; *RICTOR* non-amplified/wild-type = gray square; Gene mutation = blue square; Gene amplification = red square. Bottom Panels – Enrichment probability for co-mutant genes with *RICTOR.* For BATTLE-2 the MAPK pathway is the combination of six genes (*BRAF, KRAS, MAP2K1, MAP2K2, NF1, NRAS*). For TCGA the MAPK pathway is the combination of seven genes (*BRAF, KRAS, MAP2K1, MAP2K2, NF1, NRAS, RIT*). We consider MAPK pathway as mutation when any of these genes are mutated.

We analyzed 92 LUAD tumor biopsies obtained from BATTLE-2 chemo-refractory patients, with matching targeted genomic next-generation sequencing (NGS) and expression profiling analyses [17]. Figure 1A illustrates *RICTOR* gene alterations in BATTLE-2. Similar frequencies were found in the TCGA, which includes a majority of early stage resected LUAD [4, 18] (Figure 1A). *RICTOR* mutations in BATTLE-2 cases were all non-synonymous missense, not previously identified in public datasets, and were never concomitant with gene amplification. Since RICTOR protein structure is not fully characterized, any *in silico* prediction for the functional significance of these mutations is likely inaccurate; thus, we excluded gene mutations from our correlative studies with expression, clinical outcome, and *in vitro*/*in vivo* assays.

We evaluated the relationship between *RICTOR* amplification and gene expression. Amplification carried a moderate increased expression in BATTLE-2 (median: 6.50 (ranges: 5.71–8.71) in amplified vs 6.32 (ranges: 5.34–7.84) in non-amplified; Wilcoxon test *p* = 0.4, FC = 1.19); and a significant increase in TCGA (median: 10.64 (ranges: 9.48–13.03) in amplified vs 9.71 (ranges: 7.20–11.82) in non-amplified; Wilcoxon test *p* < 0.001, FC = 2.04). Of interest, our survey of 176 lung cancer cell lines, including all histologic subtypes using the Cancer Cell Lines Encyclopedia (Broad Institute - http://www.broadinstitute.org/ccle), further underscored the relationship between *RICTOR* mRNA levels and gene copy numbers (Supplementary Figure 1).

Next, we determined the effects of *RICTOR* mRNA expression on clinical outcome of LUAD. For this analysis, in addition to BATTLE-2 and TCGA, we examined 131 early stage surgically resected LUADs from our independent PROSPECT cohort. As shown in Figure 1B, using Cox proportional hazards model, we found the level of *RICTOR* mRNA expression to significantly portend worse overall survival in advanced LUAD BATTLE-2 patients (OS, hazard ratio [HR]: 1.73, 95% confidence interval [CI]: 1.23–2.42, *p* = 0.0015). We also detected a similar effect in our resected LUAD PROSPECT (OS, HR: 1.54, 95% CI: 1.03–2.29, *p* = 0.0337); however, no significance was seen in patients from TCGA.

### Co-mutational landscape in RICTOR-altered cases shows significant enrichment in MAPK key upstream genes in both advanced and early LUAD

We comparatively explored the co-mutational landscape of *RICTOR-*altered cases (including amplification and mutations) in BATTLE-2 and TCGA (Figure 1C, top panels). Because of our central focus on advanced LUAD, we developed our comparative studies based on genes annotated in the BATTLE-2, in which the targeted NGS encompassed 280 cancer genes. High frequency of *TP53* changes were consistently found in both datasets. Very low frequency of *STK11* were found in both, indicating co-exclusivity of *RICTOR* and *STK11* alterations. Interestingly, in BATTLE-2 about 81.2% of *RICTOR* alterations were accompanied by genomic hits of either *KRAS* (37.5%) or *EFGR* (38%), compared to the TCGA where about only 50% co-existed with *RAS* (*KRAS* (29%), *B-RAF* (16%), *N-RAS* (3%) or *EGFR* (16%)). High frequency of co-mutations was found with *NF1* (BATTLE-2: 25%, TCGA: 29%). NF1 (Neurofibromin 1) acts as a tumor suppressor by turning off *KRAS* and its mutation is often mutually exclusive with *KRAS*, as it imposes a functional activation of the RAS pathway.

To measure the association of genomic alterations of *RICTOR* with other genes, we assessed the enrichment probability (EP), which quantifies the probability of specific patient cohorts to exhibit higher/lower rates of enrichment for other specific mutations, and we considered >50% as cut-off for significant enrichment. As shown in Figure 1C bottom panels, altered *RICTOR* was significantly enriched in *NF1* mutations similarly in advanced and early disease. Enhanced enrichment in *EGFR* and *KEAP1* mutations were observed in advanced RICTOR altered LUAD, while *KRAS* mutations did not. We next assessed enrichment in signaling pathways defined as a combination of 3 or more nodal genes that were annotated in the *RICTOR* BATTLE-2 cohort. Using a combination of six genes (Figure 1C – bottom left panel) in the BATTLE-2 and seven (Figure 1C – bottom right panel) in the TCGA we found the MAPK pathway to be highly enriched in both settings. Importantly, we carried the analysis further by either including (data not shown) or excluding (data shown in Figure 1C) *EGFR* alterations, and in both cases *MAPK* pathway was enriched. Interestingly, we also observed qualitative differences in *RICTOR* and *KRAS* co-mutational landscapes, with only *RICTOR* amplifications associated with mut*KRAS* in advanced stage as opposed to the early stages where both *RICTOR* mutations and amplification were broadly distributed with mut*KRAS*. In addition, altered *RICTOR* was not preferentially associated with specific hot spot mutations of *EGFR*, *KRAS*, *BRAF*, or *NF1*.

### Compensatory MAPK signaling activation following RICTOR inhibition in mutKRAS settings

To study the oncogenic effects imposed by RICTOR, we established a RICTOR cell panel utilizing a SNP-array to detect *RICTOR* copy number variations (CNVs) across 57 NSCLC cell lines, and selected 7 *RICTOR* amplified and 5 *RICTOR* non-amplified lines spanning diverse secondary mutational backgrounds, including *KRAS*, *EGFR* and *ALK* gene alterations (Supplementary Figure 2 and Supplementary Table 1). Quantification of RICTOR protein in our panel showed, as expected, an overall higher expression in amplified lines compared to the non-amplified cells (Supplementary Figure 3). Additionally, increased mTORC2 activity markers were seen in our amplified cells, with an overall increase in p-PKCα S657 levels and elevated p-NDRG1 T346, a recognized surrogate marker for SGK1 (Supplementary Figure 3) [19].

**Figure 2:**
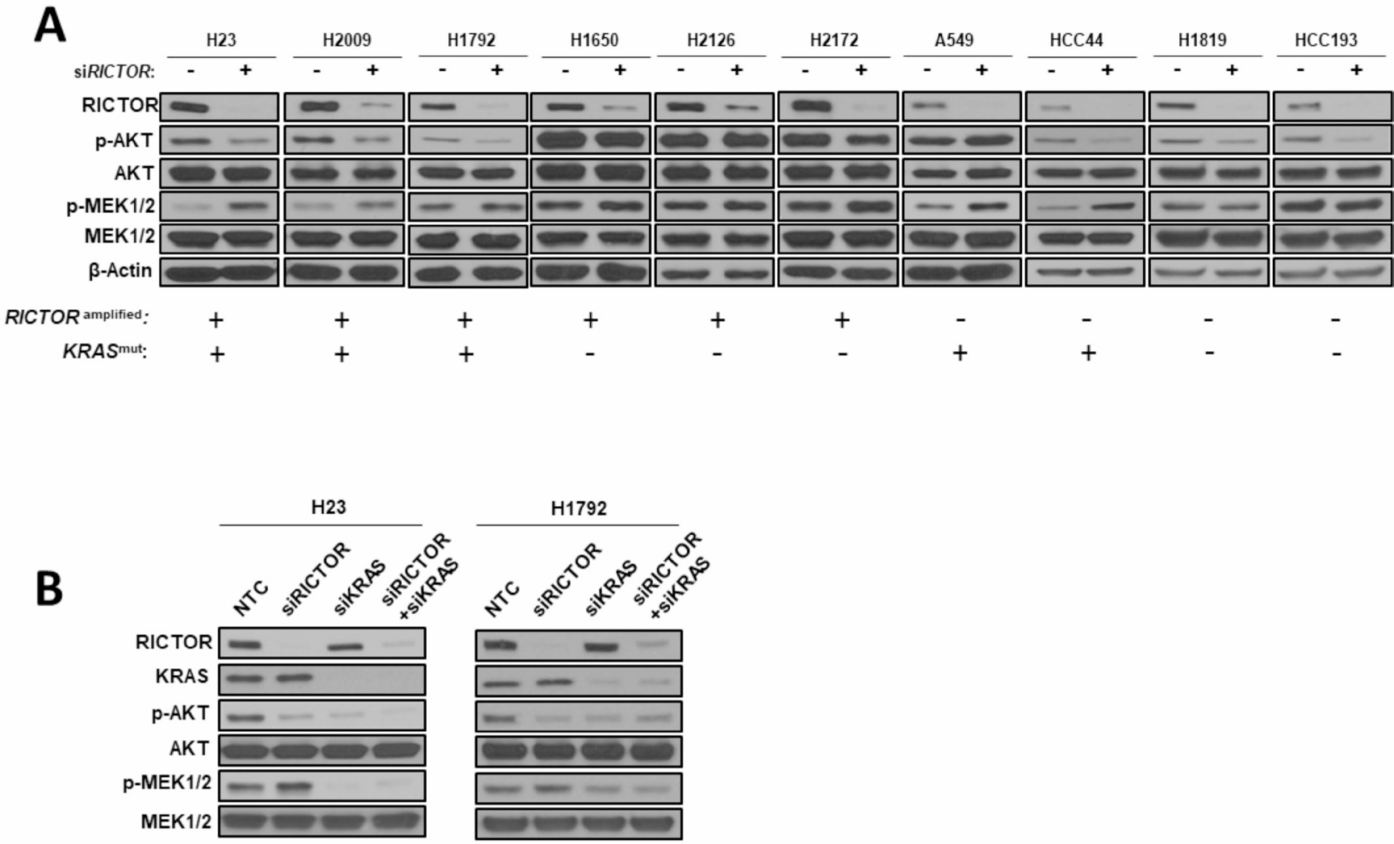
Compensatory MAPK signaling activation following *RICTOR* knockdown in *KRAS* mutant settings (**A**) A panel of 6 *RICTOR* amplified and 4 non-amplified NSCLC cell lines that are *KRAS* wild-type or mutant were transfected with siRNAs specific for *RICTOR* or scrambled negative control for 72 hours and cell lysates were analyzed by Western blotting for the specified proteins. (**B**) H23 and H1792 cells were transfected with siRNAs specific for *RICTOR*, *KRAS*, or scrambled negative control for 72 hours and cell lysates were analyzed by Western blotting for the specified proteins.

Intrigued by the co-mutational enrichment in upstream MAPK effectors, we explored the interplay signaling of RICTOR with the PI3K/AKT/mTOR, and the RAS/RAF/MEK pathways. Knockdown studies via siRNA *RICTOR* were conducted in cell lines harboring either *RICTOR* amplifications and/or *KRAS* mutations. As expected, *RICTOR* knockdown translated into a reduction in full activation of AKT S473 (p-AKT), a hallmark of active mTORC2 (Figure 2A). Of note, p-AKT levels were not reduced following RICTOR inhibition in H1650 (*EGFR, PIK3CA, PTEN* mutant) and H2126 (*LKB1* mutant), likely due to their mutational PI3K/AKT/mTOR landscape.

Furthermore, we observed a compensatory increased activation of the MAPK pathway, as shown by the elevated levels of phosphorylated MEK (p-MEK1/2) in *RICTOR* amplified cell lines (H23, H2009, H1792) harboring mut*KRAS*. To confirm whether this compensatory rebalance occurs specifically in a *KRAS* mutant setting, we performed knockdown experiments in three *RICTOR* amplified but *KRAS* wild-type cell lines (H1650, H2126, H2172), in which we could not detect increased p-MEK1/2 levels upon *RICTOR* blockade. Interestingly, a similar compensatory MAPK pathway activation was seen in *RICTOR* non-amplified but mut*KRAS* cell lines (A549, HCC44), but not in *KRAS* wild-type cell lines (H1819, HCC193) (Figure 2A).

To test whether the increased MAPK compensatory mechanism results from a unique interplay of RICTOR in mut*KRAS* backgrounds, we performed double knockdown via siRNA of *RICTOR* and *KRAS* alone, or in combination. As seen in Figure 2B, in *RICTOR* amplified mut*KRAS* cell lines (H23, H1792), siRNA *RICTOR* resulted in an elevated activation of p-MEK1/2, whereas si*KRAS* alone reduced the p-MEK1/2 levels and hence decreased MAPK pathway activity. When achieving concomitant blockade of both *RICTOR* and *KRAS*, no increase in p-MEK1/2 levels was observed, confirming our initial hypothesis.

### RICTOR blockade affects survival and tumorigenic properties of LUAD *in vitro* and *in vivo*

**Figure 3:**
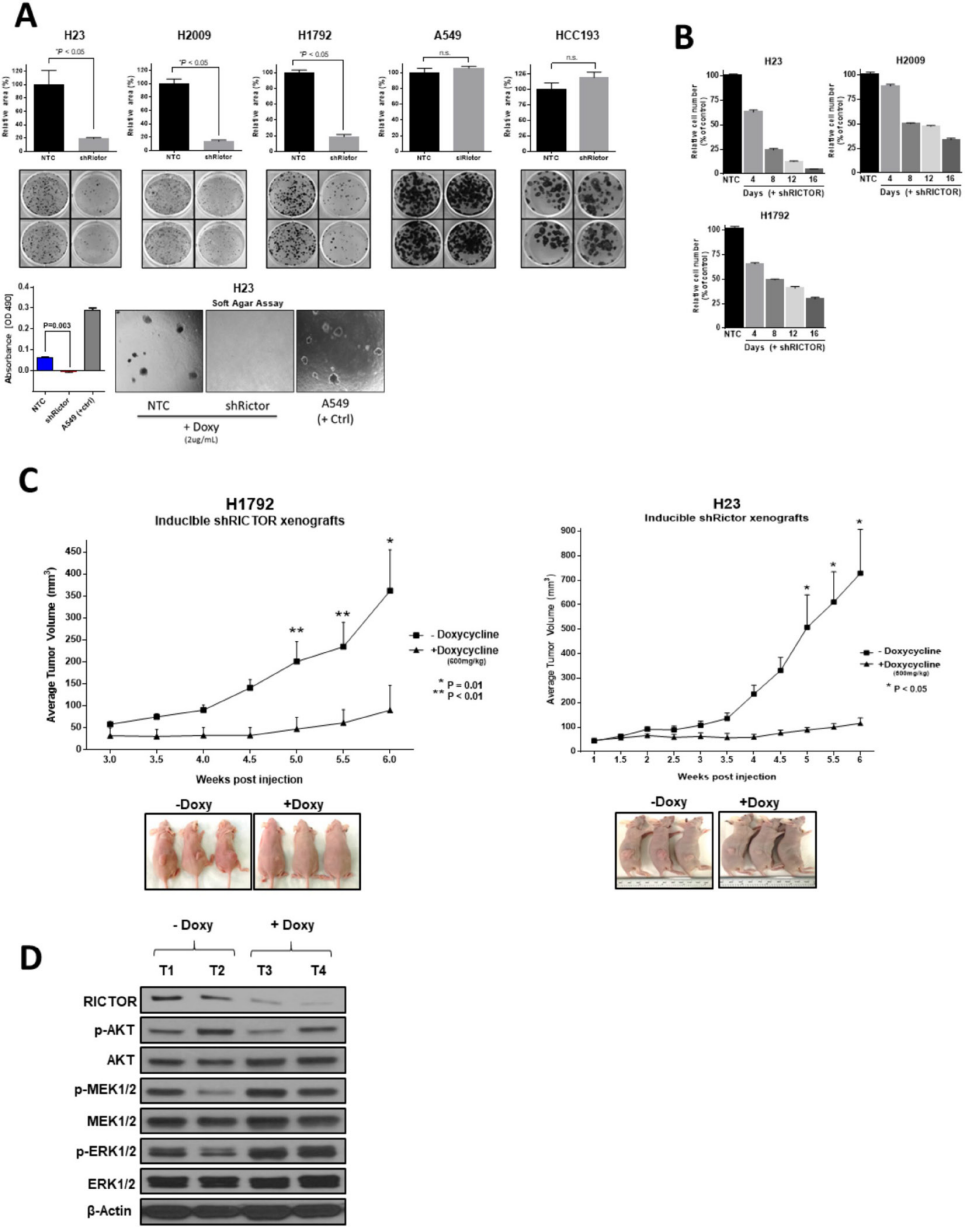
RICTOR enhances the malignant phenotype of NSCLC *in vitro* and *in vivo* (**A**) (Top) Colony formation assay of 3 *RICTOR* amplified cell lines (H23, H2009, H1792) and 2 non-amplified cell lines (A549, HCC193) comparing *RICTOR* knockdown to non-targeting control. Data are graphed as the mean percentage ± percent SD. (Bottom) Anchorage-independent growth assay in soft agar of stably transduced H23 cell line with *RICTOR* knockdown (A549 serves as positive control). ^*^P < 0.05; n.s. = not significant. (**B**) Quantification of the cell number counts of sh*RICTOR* cells relative to NTC cells at the indicated time points following doxycycline treatment. Complete cell counts were performed following 4, 8, 12, 16 days of incubation and shown as percentage relative to NTC. (**C**) Athymic nude mice were inoculated with H1792 or H23 cell lines and were fed either doxycycline (+Doxy, 600mg/kg) or control diet (–Doxy). Tumor volumes were measured twice weekly. Data points are presented as the mean tumor volume ± SEM. Representative images of xenograft tumors from each group before tumor harvesting are shown. ^*^P = 0.01; ^**^P < 0.01. (**D**) Lysates extracted from H1792 tumor xenografts were subjected to Western blot analysis with the indicated antibodies.

To determine the phenotypic consequences of *RICTOR* knockdown *in vitro* and *in vivo*, we established stably transduced doxycycline (doxy)-inducible shRNA *RICTOR* knockdown cells that either possess (H23, H2009, H1729) or lack (A549, HCC193) amplification of *RICTOR,* or used siRNA in cells challenging to transduce. *RICTOR* knockdown significantly reduced colony formation measured at three weeks in all 3 *RICTOR* amplified lines compared to non-targeting controls (NTC) (*P* < 0.05) (Figure 3A, top). Similar effects were not detected in the non-amplified cell lines A549 and HCC193, suggesting that *RICTOR* amplification may provide a survival advantage to LUAD cells. Additionally, anchorage-independent growth assay testing the transformative ability of *RICTOR* in H23 cells, using doxy-regulated sh*RICTOR* or NTC, showed complete abrogation of colony formation upon *RICTOR* downregulation, suggesting *RICTOR* contributes to the maintenance of transformative properties in cells (Figure 3A, bottom).

The contribution of amplified RICTOR to cell proliferation was assessed in H23, H2009, and H1792 cells cultured with or without *RICTOR* knockdown for 4, 8, 12, and 16 days (Figure 3B). In all 3 lines, reducing RICTOR levels resulted in markedly reduced total cell numbers as early as 4 days, and decreased the cell numbers by over 75% by day 16, yielding similar results to the colony formation assay.

Further, we investigated the role of RICTOR *in vivo* by using inducible *shRICTOR* H1792 and H23 cell murine xenografts. Continuous induction of *RICTOR* knockdown for 6 weeks translated to a significant reduction of xenograft tumor growth compared to control groups (*P* < 0.05) (Figure 3C). Molecular signaling patterns, assessed using total protein lysates from both doxycycline-treated and control H1792 xenografts, confirmed our *in vitro* results. As shown in Figure 3D, RICTOR reduction produced an overall decrease in p-AKT levels and a compensatory increase in p-MEK1/2 and its downstream target p-ERK1/2.

### RICTOR knockdown enhances the pharmacologic efficacy of MAPK pathway inhibition in RICTOR/KRAS-altered NSCLC cell lines

**Figure 4:**
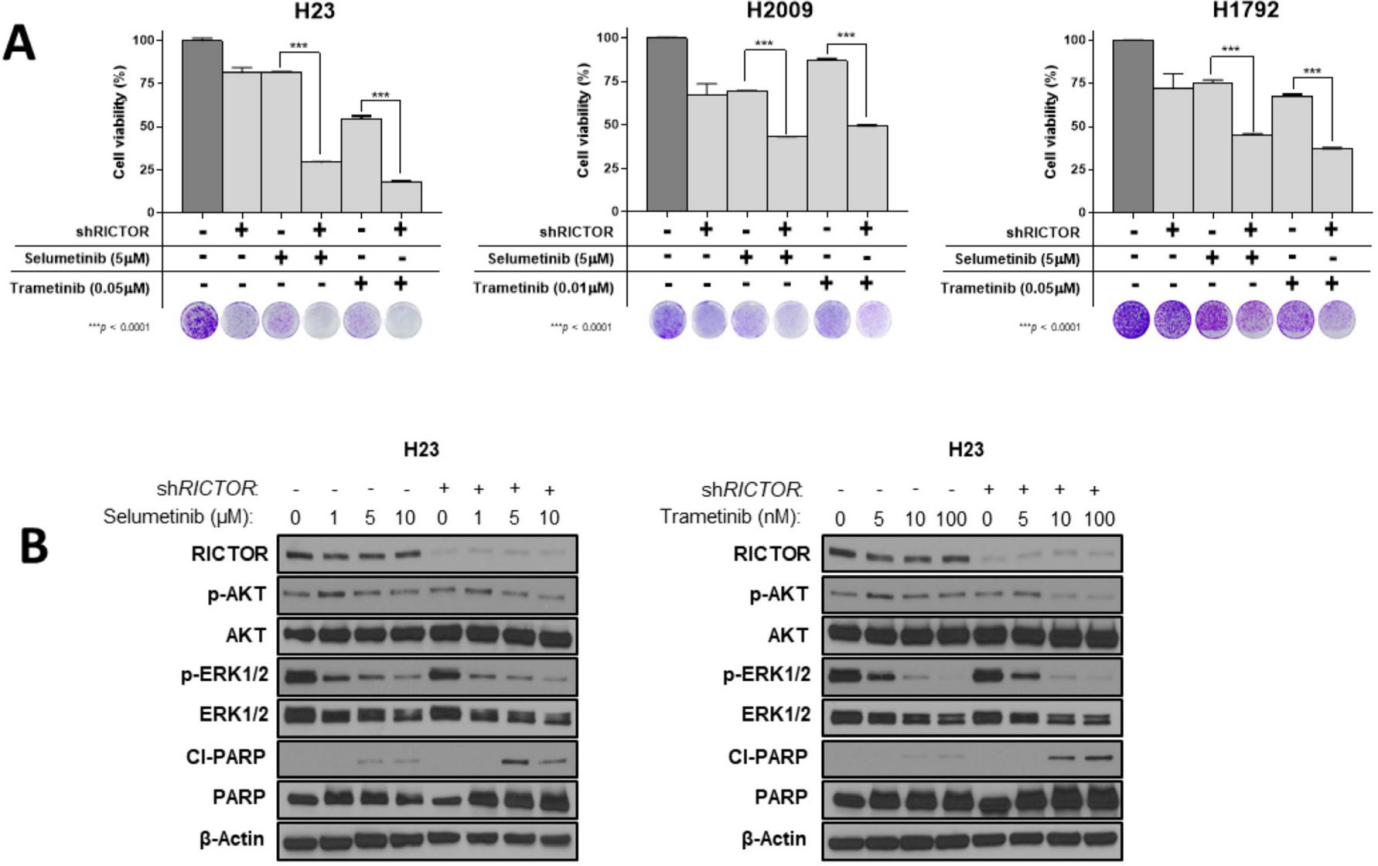
*RICTOR* knockdown enhances the pharmacologic efficacy of MAPK inhibition in *RICTOR*/*KRAS*-altered settings (**A**) Inducible sh*RICTOR* amplified NSCLC cell lines were cultured in the presence or absence of 2 μg/mL doxycycline to induce sh*RICTOR* knockdown, alone or in combination with either selumetinib (AZD6244, 5 μM) or trametinib (0.01 μM or 0.05 μM). After 7 to 10 days of treatment, cell viability was measured by MTT assay and compared between sh*RICTOR* alone or in combination with MEK1/2 inhibitors. Separate wells were stained with crystal violet on the same day to visualize and complement cell viability data. Data are graphed as the mean percentage ± percent SD. ^***^P < 0.0001. (**B**) Western blot analysis of inducible H23 cell line treated with increased doses of selumetinib (left, 1, 5, 10 μM) or trametinib (right, 5, 10, 100 nM) alone or in combination with 2 μg/mL doxycycline to induce sh*RICTOR* knockdown for a total 6 days.

To test for a unique therapeutic vulnerability offered by the dynamic interplay between RICTOR and KRAS/MAPK axis, we evaluated the pharmacological blockade of the MEK-ERK signaling pathway alone or in combination with genetic abrogation of *RICTOR,* in defined *KRAS* co-mutational settings *in vitro*. We first tested the sensitivity to two currently available allosteric MEK1/2 inhibitors (MEKi), selumetinib (AZD6244) and trametinib (GSK1120212), in *RICTOR* amplified mut*KRAS* cell lines (Figure 4A). All three cell lines displayed intrinsic resistance (>50% viability) to selumetinib (5 μM) or trametinib (0.01 μM or 0.05 μM) alone. However, the concomitant abrogation of *RICTOR* expression by shRNA resulted in a significant reduction in cell viability compared to either inhibitor alone (*P* < 0.0001). Molecularly, as shown in Figure 4B, MEKi alone or combinatorial intervention with MEKi - sh*RICTOR* suppressed AKT and MAPK signaling pathways (seen by reduced p-AKT and p-ERK levels) in a dose-dependent manner. Importantly, only the dual strategy produced increased cleaved PARP (cl-PARP) levels, suggesting that the decreased viability ascertained by MTT (Figure 4A) results from apoptosis.

### Combined mTORC1/2 and MEK inhibition is an effective therapeutic approach in RICTOR/KRAS-altered settings and results in synergistic anti-tumor effects

**Figure 5:**
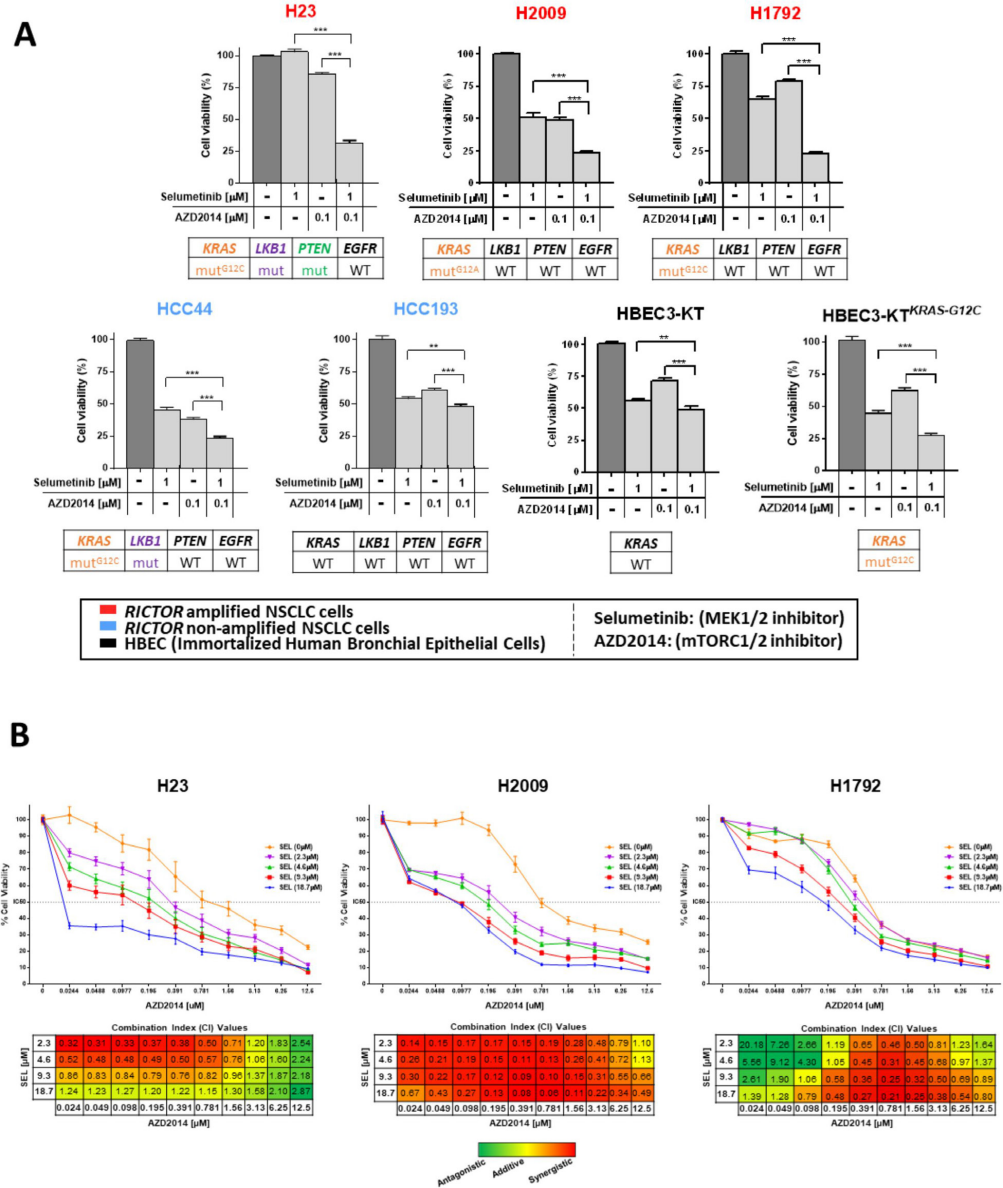
Combined mTORC1/2 and MEK inhibition is an effective therapeutic approach in *RICTOR*/*KRAS*-altered settings and results in synergistic anti-tumor effects (**A**) Five NSCLC cell lines (3 *RICTOR* amplified (red), 2 *RICTOR* non-amplified (blue) and two immortalized human bronchial epithelial cell lines (HBECs, black) were treated with DMSO (control), selumetinib (1 μM), AZD2014 (0.1 μM), or the combination selumetinib (1 μM) with AZD2014 (0.1 μM). Mutation status of *KRAS*, *LKB1, PTEN,* and *EGFR* are shown below each cell line. After 72 hours of treatment, cell viability was compared to control DMSO treated cells and measured by MTT assay. Data are graphed as the mean percentage ± percent SD. ^**^P < 0.01; ^***^P < 0.0001. (**B**) NSCLC cells were incubated with increasing concentrations of AZD2014 (0.024–12.5 μM) and a fixed dose of selumetinib (0, 2.3, 4.6, 9.3, or 18.7 μM) for 96 hours. Controls were treated with DMSO only. Cell viability was analyzed by MTS assay. Data are graphed as the mean percentage ± percent SD. Combination index (CI) values were calculated using ComboSyn software (ComboSyn Inc, Paramus, NJ). The CI parameters used were: CI = 0–0.9, synergism; CI = 0.9–1.1, additive effect; CI > 1.1, antagonism.

Currently, pharmacological inhibition of RICTOR is not available, however a dual catalytic mTORC1/2 inhibitor (AZD2014) is currently in phase II clinical trials offering the opportunity to test our dual pathway approach with a clinically relevant compound. *RICTOR* amplified or non-amplified LUAD cell lines, carrying various mutational backgrounds affecting the PI3K/AKT/mTOR and/or KRAS/MAPK pathways, were exposed to selumetinib and AZD2014 alone or in combination (Figure 5A). In H23, H2009, and H1792 cell lines (*RICTOR* amplified, mut*KRAS*), concomitant targeting of mTORC1/2 and MEK resulted in a 75% reduction of cell viability. Of note, H23 also harbors *LKB1* and *PTEN* mutations, likely accounting for enhanced intrinsic resistance to either single agent compared to H2009 or H1792.

Interestingly, HCC44, carrying mut*KRAS* but not *RICTOR* amplification, exhibited increased sensitivity to single agents selumetinib and AZD2014 compared to cell lines harboring both *RICTOR* and *KRAS* alterations, while remaining more sensitive to dual inhibition. In contrast, the HCC193 cell line, wild-type across many key drivers, showed relative resistance to either agent alone or the combination (>50% cell viability). To further confirm that this dual pathway inhibition is most effective in a mut*KRAS* setting, we used isogenic human bronchial epithelial cell lines (HBECs) previously described [20, 21], that either carry *KRAS* wild-type (HBEC3-KT) or mut*KRAS* G12C (HBEC3-KT*^KRAS-G12C^*). HBEC3-KT*^KRAS-G12C^* cells exhibited significantly higher sensitivity (~75% reduction in cell viability) to the dual blockade combination compared to their *KRAS* wild-type counterpart (>50% viability) (Figure 5A).

In addition, we assessed whether the combination therapy of both drugs resulted in synergistic, additive, or antagonistic effects across a range of therapeutic doses by MTS assay. H23, H2009, H1792 cell lines (*RICTOR* amplified, mut*KRAS*) were treated with various concentrations of AZD2014 (0.024–12.5 μM) and a fixed set of selumetinib doses (2.3, 4.6, 9.3, or 18.7 μM) for 96 hours (Figure 5B, top). In all three representative cell lines, optimal drug dose combinations that impose synergistic effects were found (Figure 5B, bottom). H23 showed the highest level of synergism in the range of AZD2014 (0.024–0.781 μM) combined with selumetinib (2.3 or 4.6 μM), whereas increasing the AZD2014 and selumetinib combination doses resulted in a loss of synergy and caused an additive or antagonistic effect. In H2009, we found consistent synergism across most of the combination dose ranges; and in H1792 the synergistic and/or additive effects were observed in the range of AZD2014 (0.195–6.25 μM) combined with selumetinib (2.3–18.7 μM).

### Dual mTORC1/2 and MEK1/2 pathway inhibition results in the strongest anti-tumor effect *in vivo*

**Figure 6:**
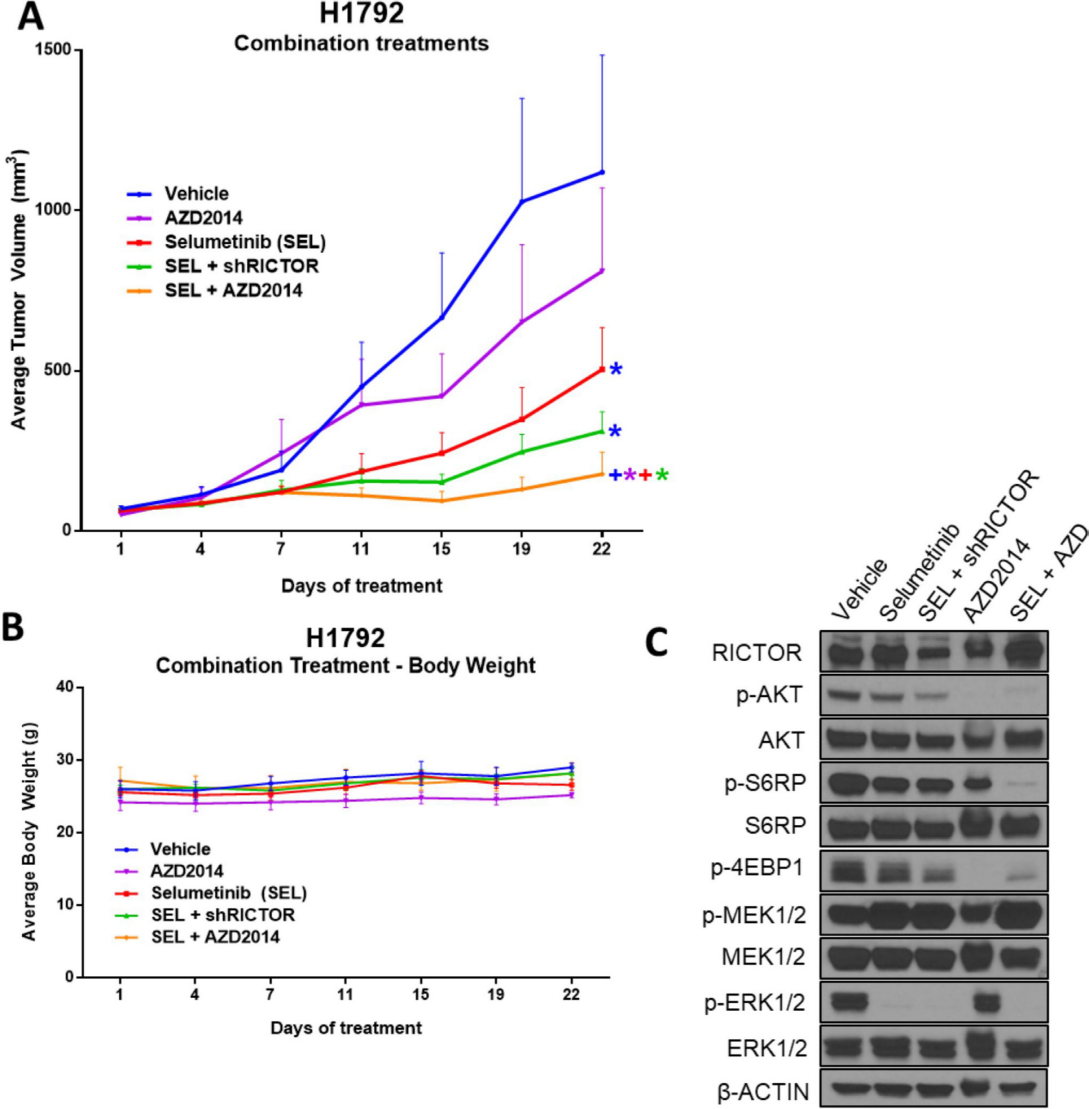
Dual mTORC1/2 and MEK1/2 pathway inhibition results in the strongest anti-tumor effect *in vivo* (**A**) Athymic nude mice were inoculated subcutaneously with the inducible H1792 sh*RICTOR* cell line. Once tumor volumes reached an average of 100 mm^3^, mice were randomized into five treatment arms: vehicle (1% tween-80, bid), selumetinib (25 mg/kg, bid), selumetinib (25 mg/kg, bid) + doxycycline feed (600 mg/kg), AZD2014 (15 mg/kg, qd), and selumetinib + AZD2014 (equivalent dosages used as per individual inhibitor treatments). Treatments were administered via oral gavage daily for a total of 22 days. Tumor volumes were measured twice weekly, and data points are presented as the mean tumor volume ± SEM. Colored asterisks represent significant difference of that treatment from a different treatment resembled by its respective line color. (^*^P < 0.05; ^+^*P* < 0.01). (**B**) Average body weight of mice is displayed for each treatment arm, and measured twice weekly. (**C**) Western blot analysis showing the levels of indicated proteins in tumor lysates harvested 3 hours after last drug treatment on day 22.

To determine whether the synergistic effects seen *in vitro* translated into anti-tumorigenic effects *in vivo*, we utilized inducible sh*RICTOR*-H1792 cells (*RICTOR* amplified, mut*KRAS*) to establish tumor xenografts in mice. Mice receiving selumetinib with AZD2014 exhibited the greatest anti-tumor effects compared to each single-agent treatment group; and while a robust anti-tumor effect was also achieved in the selumetinib + sh*RICTOR* (+Doxy) arm, dual MEKi and mTORC1/2 treatment achieved a significantly pronounced reduction in tumor volume (Figure 6A). Interestingly, in concordance with our *in vitro* H1792 cell viability MTT data (Figure 5A), selumetinib treatment alone resulted in an increased reduction of cell viability/tumor growth compared to single agent AZD2014 treatment, suggesting that this particular mut*KRAS* setting (*KRAS^G12C^)* is more sensitive to MEK1/2 inhibition than mTORC1/2, despite *RICTOR* amplification. Of note, no significant loss of body weight or visible signs of declined health were witnessed during the course of treatment (Figure 6B).

Moreover, molecular pathway analysis from each treatment arm confirmed the engagement of each inhibitor on its target (Figure 6C). The treatment groups incorporating selumetinib effectively blocked downstream p-ERK1/2 signaling, AZD2014 had a marked reduction in downstream mTORC1/2 effectors p-AKT, p-S6RP and p-4EBP1, and blockade of RICTOR via shRNA + doxycycline reduced total RICTOR protein levels. Collectively, these findings suggest that AZD2014, in combination with selumetinib, results in significant anti-tumor activity of *RICTOR*/*KRAS-*altered LUAD, through simultaneous blockade of mTORC1/2 and MEK pathways.

## DISCUSSION

The strengths of our study reside in providing a broader analysis of RICTOR's clinical significance by surveying 3 independent cohorts representing the clinical evolution of LUAD, from early to advanced; in conducting phenotypic and pharmacologic studies in a large carefully chosen panel of NSCLC cell lines harboring diverse driving genetic makeups; and in providing *in vivo* confirmation of clinically applicable findings.

Our first novel finding is that while *RICTOR* genetic alterations have similar frequency in early and late stage, *RICTOR* mRNA expression significantly portends worse survival in advanced refractory disease, but not in early untreated settings. Interestingly, a similar effect was observed in our PROSPECT cohort: untreated surgically resected LUAD more clinically advanced than the TCGA cohort. These results support a co-oncogenic role for *RICTOR* as the disease advances and the tumor survival becomes intrinsically dependent on molecular mechanisms sustaining metabolic overdrive. An indirect supporting evidence for RICTOR dependence is perhaps the correlation between *RICTOR* expression – tumor stage and tumor size found in our multivariate analysis of overall survival in PROSPECT (data not shown). We were unable to assess the full prognostic implication of *RICTOR* genetic alterations, limited by the relatively small number of genetic events in each cohort and by the current lack of functional significance for gene mutations. However, the correlation between amplification and increased mRNA expression suggests that amplification could be driving a worse outcome in a subset of cases. Our results also suggest that additional mechanisms might be contributing to RICTOR's increased expression when gene amplification is absent; however, our study did not further investigate this. Analyses in larger cohorts are warranted to provide more conclusive information on RICTOR's prognostic role and potential biomarker development.

A study by *Cheng et al.* reported *RICTOR* amplification in a distinct subset of lung cancer patients, a portion of which harbored amplification as the sole potentially actionable target [22]. In concordance with our current *in vitro* clonogenic and *in vivo* data, the study by *Cheng et al.* suggested an oncogenic role of RICTOR, and proposed dual mTORC1/2 inhibition as an optimal therapeutic approach in *RICTOR* amplified lung cancers. While a dual mTORC1/2 intervention in a mono-oncogenic *RICTOR* amplified setting has a clear value, our findings suggest that this treatment strategy may not be as effective in NSCLC where other genomic aberrations are present alongside *RICTOR* amplification. In fact, we provide first time evidence that the co-mutational landscape of *RICTOR* genetic alterations is significantly enriched with genetic alterations in nodal effectors of the MAPK pathway, in both early and late stage LUAD. Our findings have a two-fold significance. First, it appears that as the disease progresses, genomic altered *RICTOR* switches from mono-oncogenic to co-oncogenic, specifically with *KRAS* and *EGFR*, suggesting that mono-oncogenic *RICTOR* may not carry enough power to drive tumors to advanced stage. Second, the significant enrichment in *NF1* mutations, in early as well as advanced disease, further underscores the functional interplay of RICTOR and the KRAS/MAPK axis in a subset of cases. *NF1* mutations may provide an intrinsically hyperactive KRAS/MAPK axis in a subset of *RICTOR*/*EGFR* co-mutant settings, further supporting the functional partnership with the MAPK axis. The relatively small number of *RICTOR*-altered cases is limiting our studies, and while the thematic concordance is found between TCGA and BATTLE-2 for co-mutational patterns, spanning over 322 LUADs, we recognize future larger studies are needed.

Our *in vitro* and *in vivo* data provide functional support to our co-mutational genetic findings by conclusively demonstrating that RICTOR blockade results in a compensatory activation of the MAPK pathway, specifically in *KRAS* co-mutational settings. Importantly, we show that in, both, amplified and non-amplified NSCLC cells, *RICTOR* knockdown increases p-MEK levels only when the cells harbor mut*KRAS*. We confirmed, *in vitro*, the dependence on mutKRAS to elicit compensatory activation of the MAPK pathway once RICTOR is blocked. Though the precise mechanism linking this unique interplay in the context of our tested cells needs to be elucidated, one may suspect that given the increased findings of RICTOR's non-canonical functionalities (independent activity of the mTOR/AKT pathway), RICTOR could be directly or indirectly eliciting repressive signals on the KRAS pathway such that, when RICTOR is blocked, the mutKRAS cells compensate survival mechanisms via upregulation of the MAPK pathway mediated by mutKRAS. This survival advantage could be an adaptation specific to mutKRAS and not wild-type NSCLC cells. We therefore exploited this survival mechanism as a therapeutic vulnerability with a dual pathway inhibitory approach using a catalytic mTORC1/2 inhibitor (AZD2014) and an allosteric MEK1/2 inhibitor. Our results suggest this combination renders a highly synergistic anti-tumor effect at specific dose ranges in *RICTOR*/*KRAS-*altered NSCLC cells, both *in vitro* and *in vivo*. This is an important finding as it may offer therapeutic options for mut*KRAS* tumors, representing 25–30% of LUAD, known to carry resistance to chemo- and targeted therapies [23, 24]. To date, mut*KRAS* tumors remain therapeutic orphan targets, as direct targeting of aberrant KRAS activation has been unsuccessful despite significant research efforts [25].

Various targeted therapies have been developed to inhibit the PI3K/AKT/mTOR and KRAS/MAPK oncogenic signaling pathways [26, 27]. However, with few exceptions, the utility of these drugs and others as single agents has been disappointing to date mostly because of molecular bypass mechanisms. Evidence exists that signaling cross-talk and feedback loop mechanisms determine therapeutic inefficacy and tumor relapses, providing strong rationale for combinational therapeutic strategies. A recent *in vitro* study underscored the rationale for combined inhibition of MEK and mTOR signaling in mut*KRAS* NSCLC [28]. However, in contrast to our findings, the inhibition of mTOR appeared dominantly responsible for the majority of growth inhibition when combining mTORC1/2 inhibitor (AZD2014) with MEK1/2 inhibitor (trametenib). In addition, dual inhibition carried an improved benefit over single agents alone in mut*KRAS* lines. The difference in drug sensitivity reported here may be directly related to aberrant *RICTOR* creating an inherent rebalanced inner loop that enhances MAPK signaling. The difference could also be influenced by the MEK inhibitor (selumetinib vs. trametinib), which are known to have differing mechanisms of action. Our *in vivo* data using the H1792 xenograft model showed single agent selumetinib had enhanced anti-tumor effects compared to mTORC1/2 inhibitor AZD2014, suggesting that mut*KRAS^G12C^* is more sensitive to MEK1/2 inhibition than mTORC1/2, despite having *RICTOR* amplification (Figure 6A).

Despite differences in drugs and genetic backgrounds, there is substantial evidence supporting the dual pathway targeting combinatorial strategy proposed here. Additional studies testing the value of specific pharmacologic targeting of RICTOR may find rationale in mono-oncogenic settings. The independent mTORC2 functions of RICTOR may provide additional emphasis for developing RICTOR specific inhibitors. We believe that dual mTORC1/2 inhibitors, such as AZD2014, in combination with MEKi may provide a unique therapeutic opportunity for patients harboring mut*KRAS* and *RICTOR* amplification, preventing the feedback seen by single mTORC1 inhibitors known to induce hyper-activation of PI3K-AKT-MAPK pathways [29]. Although previous reports suggest limited clinical benefits from mTORC1/2 inhibitors, proper patient selection in lung cancer patients is needed to fully exploit this therapeutic option [30].

In conclusion, our study suggests that *RICTOR*/*KRAS*-altered LUADs offers unique therapeutic vulnerability, optimally engaged through dual mTORC1/2-MEK1/2 targeting, and underscores the importance of genomic-driven stratification to refine optimal therapeutic functional targeting in NSCLC.

## MATERIALS AND METHODS

### Clinical datasets

Three datasets were analyzed independently: the BATTLE-2 (advanced refractory LUAD - *n* = 92 cases with NGS and mRNA data; *n* = 107 LUAD with mRNA expression data), the TCGA (*n* = 230 with mutation cases, *n* = 496 with mRNA expression data), and the PROSPECT (*n* = 151 with mRNA expression data). All clinical cohorts were previously described. In all cases, bio-specimens were obtained following patient informed consent, under protocols approved by Institutional Review Boards at all participating institutions. All human studies were conducted in accordance with the Declaration of Helsinki.

### Molecular analyses

Somatic mutation, copy number (GISTIC 2.0) data for TCGA LUAD were accessed through the TCGA portal. NGS used in BATTLE-2 was performed by Foundation Medicine, Inc., as previously described [17].

### Cell lines and reagents

Cells were obtained from American Type Tissue Collection (Manassas, VA) or collaborators, authenticated via STR DNA fingerprinting at UT MD Anderson Characterized Cell Line Core. Whole genome SNP array profiling was used to determine *RICTOR* amplified (copy number variation (CNV ≥ 3.5) and non-amplified cell lines (CNV ~2) [31]. Immortalized human bronchial epithelial cells expressing wt*KRAS* (HBEC3-KT) and *KRAS* mutant with stable p53 knockdown (HBEC3-KT53KC12) lines were provided by Drs. Gazdar and Minna (UT Southwestern Medical Center, Dallas, TX). Stable *RICTOR* knockdown was developed using pTRIPZ inducible lentiviral shRNA plasmids (Rictor shRNA #RHS4696, Non-silencing shRNAmir Control (NTC) #RHS4743) (GE Dharmacon, Lafayette, CO). Targeted inhibitors AZD2014 (vistusertib), AZD6244 (selumetinib), and GSK1120212 (trametinib) were obtained from Selleck Chemicals (Houston, TX).

### Immunoblotting and antibodies

Western blotting analyses were performed on total protein lysates extracted from NSCLC cell lines. Antibodies used: RICTOR, p-RICTOR (Thr1135), p-AKT (Ser473), AKT, p-MEK1/2 (Ser217/221), MEK1/2, p-p44/42 MAPK (Thr202/Tyr204) (p-ERK1/2), ERK1/2, c-PARP, PARP, p-mTOR (S2481), mTOR, p-NDRG1 (Thr346), NDRG1, mSIN1, p-4E-BP1 (Thr37/46), KRAS, p-S6RP (S235/236), S6RP are from Cell Signaling Technologies (Danvers, MA); p-PKCα(Ser657) and β-actin-HRP are from Santa Cruz Biotechnology (Santa Cruz, CA). Immunoreactivity was visualized by Western Lightning Plus-ECL (Perkin-Elmer, Waltham, MA) and exposure to x-ray film according to manufacturer. Densitometric quantification was performed using Image Studio Lite 5.0 software (Lincoln, NE).

### siRNA knockdown

Knockdown studies of *RICTOR* or *KRAS* were performed using ON-TARGETplus SMARTpool RICTOR siRNAs (GE Dharmacon, RNAi Technologies, Thermo). ON-TARGETplus Non-targeting siRNA pools served as controls.

### Cell viability and proliferation assays

MTS assay testing cell viability were performed in octuplicate in 96-well plates with 2 μg/mL doxycycline. Combination index (CI) values were calculated using ComboSyn (ComboSyn Inc, Paramus, NJ). CI parameters: CI = 0–0.9, synergism; CI = 0.9–1.1, additive effect; CI > 1.1, antagonism. Cell proliferation was performed by seeding sh*RICTOR* cells ± doxycycline containing media. At specific time points, total cell numbers were counted using the Cellometer K2 Image Cytometer (Nexcelom, Lawrence, MA).

### Clonogenic survival assay

Cells were seeded in 6-well plates with/without 2 μg/mL doxycycline (inducible sh*RICTOR* cells) or non-targeting control or siRNA *RICTOR* for 2–3 weeks, with media change every 2–3 days. After crystal violet staining, colony areas were calculated using Image J software (NIH, Bethesda, MD). Soft agar assay was performed using Millipore's Cell Transformation Detection Assay per manufacturer recommendations (Merck KGaA, Darmstadt, Germany).

### Xenograft tumor models

Animal procedures and care were approved by the UT MD Anderson Cancer Center Institutional Animal Care and Usage Committee. Animals received humane care as per the Animal Welfare Act and the NIH “Guide for the Care and Use of Laboratory Animals”. For tumorigenicity studies evaluating the effects of *RICTOR* knockdown, sh*RICTOR* inducible cell lines were expanded, harvested, washed, pre-cooled in serum-free RPMI 1640 medium mixed 1:1 with Corning growth factor reduced Matrigel Matrix (Corning, NY). Female athymic nude mice, 6–8 weeks old, were injected subcutaneously in the flank with H1792 (2 × 10^6^) or H23 (5 × 10^6^) sh*RICTOR* cells. Mice were divided into two groups with 6 mice per arm: doxycycline feed (600 mg/kg; BioServ, Flemington, NJ) after inoculation of cells, or control group (regular feed). When tumor burden was reached, the mice were euthanized, and the tumors were extracted for protein lysate analysis. For xenograft drug studies, H1792 sh*RICTOR* cells were prepared as above and injected subcutaneously in the flanks of 6–8 week old female athymic nude mice. After the average tumor volumes reached 100 mm^3^, mice were randomized into 1 of 5 treatment arms (6 mice/arm), and the indicated treatment regimens were performed by oral gavage for 22 days. Selumetinib was obtained from Selleck Chemicals and AZD2014 was obtained from MedChem Express (Monmouth Junction, NJ). Tumor volumes and body weights were recorded twice weekly. Tumors were extracted 3 hours following the last treatment, and protein lysates were prepared. All tumors were measured twice weekly using a digital caliper, and size was calculated as (length × width^2^/2).

### Statistical analyses

The results are average of at least three experiments each performed at least in triplicate. Data obtained from cell culture assays were summarized using descriptive and inferential statistical analyses accompanied by graphs and conducted by using GraphPad Prism 6 (GraphPad Software, La Jolla, CA). Differences between groups were calculated by the *t*-test. A *P*-value < 0.05 was considered significant. Cox proportional hazard models were applied for association between mRNA expression and overall survival. For enrichment studies, we followed a Bayesian hypothesis testing framework to test whether the proportion of Gene X mutation patients is significantly higher in *RICTOR* altered patients than the proportion of patients from *RICTOR* wild-type patients. Probability was computed using Monte Carlo methods.

## SUPPLEMENTARY MATERIALS FIGURES AND TABLE



## References

[1] Chen Z, Fillmore CM, Hammerman PS, Kim CF, Wong KK (2014). Non-small-cell lung cancers: a heterogeneous set of diseases. Nat Rev Cancer.

[2] Tsao AS, Scagliotti GV, Bunn PA, Carbone DP, Warren GW, Bai C, de Koning HJ, Yousaf-Khan AU, McWilliams A, Tsao MS, Adusumilli PS, Rami-Porta R, Asamura H (2016). Scientific Advances in Lung Cancer 2015. J Thorac Oncol.

[3] Shea M, Costa DB, Rangachari D (2016). Management of advanced non-small cell lung cancers with known mutations or rearrangements: latest evidence and treatment approaches. Ther Adv Respir Dis.

[4] Cancer Genome Atlas Research Network (2014). Comprehensive molecular profiling of lung adenocarcinoma. Nature.

[5] Cox AD, Fesik SW, Kimmelman AC, Luo J, Der CJ (2014). Drugging the undruggable RAS: Mission possible?. Nat Rev Drug Discov.

[6] Papadimitrakopoulou V, Lee JJ, Wistuba II, Tsao AS, Fossella FV, Kalhor N, Gupta S, Byers LA, Izzo JG, Gettinger SN, Goldberg SB, Tang X, Miller VA (2016). The BATTLE-2 Study: A Biomarker-Integrated Targeted Therapy Study in Previously Treated Patients With Advanced Non–Small-Cell Lung Cancer. Journal of Clinical Oncology.

[7] Aimbetov R, Chen CH, Bulgakova O, Abetov D, Bissenbaev AK, Bersimbaev RI, Sarbassov DD (2012). Integrity of mTORC2 is dependent on the rictor Gly-934 site. Oncogene.

[8] Alessi DR, Pearce LR, Garcia-Martinez JM (2009). New insights into mTOR signaling: mTORC2 and beyond. Sci Signal.

[9] Sarbassov DD, Ali SM, Kim DH, Guertin DA, Latek RR, Erdjument-Bromage H, Tempst P, Sabatini DM (2004). Rictor, a novel binding partner of mTOR, defines a rapamycin-insensitive and raptor-independent pathway that regulates the cytoskeleton. Curr Biol.

[10] Ikenoue T, Inoki K, Yang Q, Zhou X, Guan KL (2008). Essential function of TORC2 in PKC and Akt turn motif phosphorylation, maturation and signalling. EMBO J.

[11] Zhipeng Z, Juan C, Jun Y, Xiaochun B (2016). Targeted Inhibition of Rictor/mTORC2 in Cancer Treatment: A New Era after Rapamycin. Current Cancer Drug Targets.

[12] Oh WJ, Jacinto E (2011). mTOR complex 2 signaling and functions. Cell Cycle.

[13] Agarwal NK, Chen CH, Cho H, Boulbes DR, Spooner E, Sarbassov DD (2013). Rictor regulates cell migration by suppressing RhoGDI2. Oncogene.

[14] Gao D, Wan L, Inuzuka H, Berg AH, Tseng A, Zhai B, Shaik S, Bennett E, Tron AE, Gasser JA, Lau A, Gygi SP, Harper JW (2010). Rictor forms a complex with Cullin-1 to promote SGK1 ubiquitination and destruction. Mol Cell.

[15] McDonald PC, Oloumi A, Mills J, Dobreva I, Maidan M, Gray V, Wederell ED, Bally MB, Foster LJ, Dedhar S (2008). Rictor and integrin-linked kinase interact and regulate Akt phosphorylation and cancer cell survival. Cancer Res.

[16] Serrano I, McDonald PC, Lock FE, Dedhar S (2013). Role of the integrin-linked kinase (ILK)/Rictor complex in TGFbeta-1-induced epithelial-mesenchymal transition (EMT). Oncogene.

[17] Frampton GM, Fichtenholtz A, Otto GA, Wang K, Downing SR, He J, Schnall-Levin M, White J, Sanford EM, An P, Sun J, Juhn F, Brennan K (2013). Development and validation of a clinical cancer genomic profiling test based on massively parallel DNA sequencing. Nature Biotechnology.

[18] Gao J, Aksoy BA, Dogrusoz U, Dresdner G, Gross B, Sumer SO, Sun Y, Jacobsen A, Sinha R, Larsson E, Cerami E, Sander C, Schultz N (2013). Integrative analysis of complex cancer genomics and clinical profiles using the cBioPortal. Sci Signal.

[19] Garcia-Martinez JM, Alessi DR (2008). mTOR complex 2 (mTORC2) controls hydrophobic motif phosphorylation and activation of serum- and glucocorticoid-induced protein kinase 1 (SGK1). Biochem J.

[20] Sato M, Vaughan MB, Girard L, Peyton M, Lee W, Shames DS, Ramirez RD, Sunaga N, Gazdar AF, Shay JW, Minna JD (2006). Multiple oncogenic changes (K-RAS(V12), p53 knockdown, mutant EGFRs, p16 bypass, telomerase) are not sufficient to confer a full malignant phenotype on human bronchial epithelial cells. Cancer Res.

[21] Ramirez RD, Sheridan S, Girard L, Sato M, Kim Y, Pollack J, Peyton M, Zou Y, Kurie JM, Dimaio JM, Milchgrub S, Smith AL, Souza RF (2004). Immortalization of human bronchial epithelial cells in the absence of viral oncoproteins. Cancer Res.

[22] Cheng H, Zou Y, Ross JS, Wang K, Liu X, Halmos B, Ali SM, Liu H, Verma A, Montagna C, Chachoua A, Goel S, Schwartz EL (2015). RICTOR amplification defines a novel subset of lung cancer patients who may benefit from treatment with mTOR1/2 inhibitors. Cancer Discov.

[23] Johnson L, Mercer K, Greenbaum D, Bronson RT, Crowley D, Tuveson DA, Jacks T (2001). Somatic activation of the K-ras oncogene causes early onset lung cancer in mice. Nature.

[24] Chan BA, Hughes BG (2015). Targeted therapy for non-small cell lung cancer: current standards and the promise of the future. Transl Lung Cancer Res.

[25] Wang Y, Kaiser CE, Frett B, Li HY (2013). Targeting mutant KRAS for anticancer therapeutics: a review of novel small molecule modulators. J Med Chem.

[26] Dienstmann R, Rodon J, Serra V, Tabernero J (2014). Picking the point of inhibition: a comparative review of PI3K/AKT/mTOR pathway inhibitors. Mol Cancer Ther.

[27] Montagut C, Settleman J (2009). Targeting the RAF-MEK-ERK pathway in cancer therapy. Cancer Lett.

[28] Broutin S, Stewart A, Thavasu P, Paci A, Bidart JM, Banerji U (2016). Insights into significance of combined inhibition of MEK and m-TOR signalling output in KRAS mutant non-small-cell lung cancer. Br J Cancer.

[29] Jokinen E, Koivunen JP (2015). MEK and PI3K inhibition in solid tumors: rationale and evidence to date. Ther Adv Med Oncol.

[30] Cheng H, Shcherba M, Pendurti G, Liang Y, Piperdi B, Perez-Soler R (2014). Targeting the PI3K/AKT/mTOR pathway: potential for lung cancer treatment. Lung Cancer Manag.

[31] Yang F, Tang X, Riquelme E, Behrens C, Nilsson MB, Giri U, Varella-Garcia M, Byers LA, Lin HY, Wang J, Raso MG, Girard L, Coombes K (2011). Increased VEGFR-2 gene copy is associated with chemoresistance and shorter survival in patients with non-small-cell lung carcinoma who receive adjuvant chemotherapy. Cancer Res.

