# Supplementary material for: Concomitant targeting of the mTOR/MAPK pathways: novel therapeutic strategy in subsets of RICTOR/KRAS-altered non-small cell lung cancer
Source: Oncotarget. 2018 Sep 21;9(74):33995–4008. doi: 10.18632/oncotarget.26129 (PMC6188056; doi:10.18632/oncotarget.26129)
Supplement: Supplementary file 1 [file oncotarget-09-33995-s001.pdf]

Concomitant targeting of the mTOR/MAPK pathways: novel therapeutic strategy in subsets of *RICTOR/KRAS*-altered non-small cell lung cancer

SUPPLEMENTARY MATERIALS

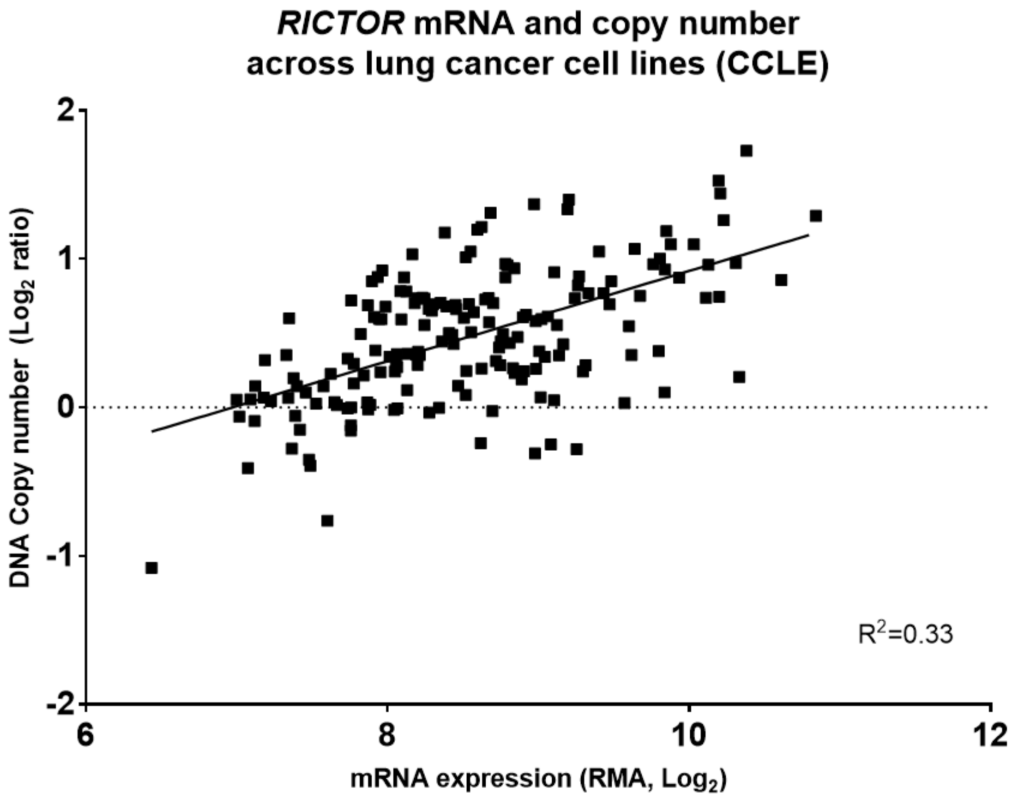

Supplementary Figure 1: Correlation of *RICTOR* mRNA expression across lung cancer cell lines from Cancer Cell Lines Encyclopedia from Broad Institute - http://www.broadinstitute.org/ccle.

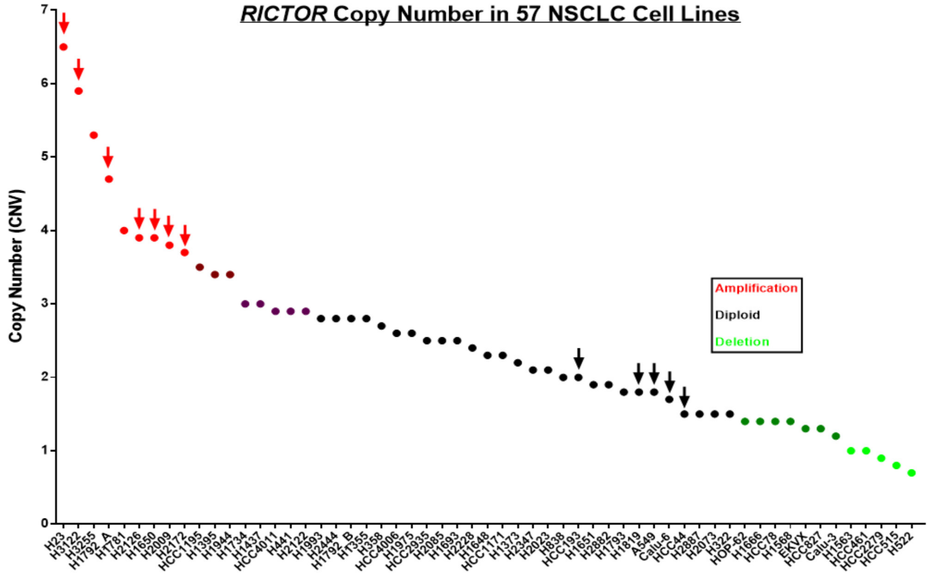

Supplementary Figure 2: *RICTOR* copy number levels across 57 NSCLC cell lines. Whole genome single nucleotide polymorphism (SNP) array profiling was obtained for 57 NSCLC cell lines to determine *RICTOR* amplified (copy number variation (CNV ≥ 3.5) and non-amplified cell lines (CNV ~2). Seven *RICTOR* amplified cell lines (red arrows) and five *RICTOR* non-amplified cell lines (black arrows) were selected. Copy number variation (CNV) values: Red = amplified; black = diploid; green = deletion.

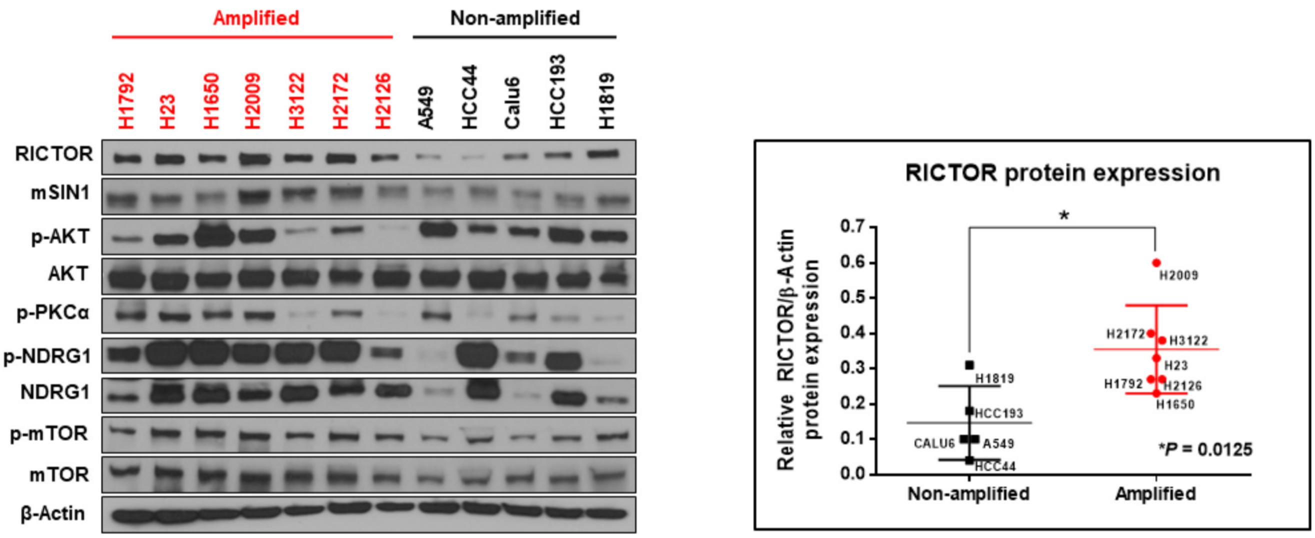

Supplementary Figure 3: Comparison of signaling and *RICTOR* expression in *RICTOR*-amplified versus non-amplified cell lines. (Left) Cell lysates from 12 NSCLC cell lines (amplified or non-amplified for *RICTOR*) were examined by Western blotting. Total and phospho-specific antibodies used were for levels of RICTOR, mSIN1, p-AKT (S473), AKT, p-PKCα (S657), p-NDRG1 (T346), NDRG1, p-mTOR (S2481), mTOR, and β-Actin as loading control. (Right) Quantification of relative RICTOR/β-Actin protein expression from densitometric analysis of western blot panel. $^{*}P = 0.01$.

Supplementary Table 1: Mutational profile of *RICTOR* cell line panel

	Cell line	*KRAS*	*EGFR*	*STK11*	*PIK3CA*	*PTEN*	*EML4/ALK*
***RICTOR* amplified**	**H2172**	WT	WT	WT	WT	WT	WT
	**H2126**	WT	WT	Mut	WT	WT	WT
	**H23**	mut - G12C	WT	Mut	WT	Mut	WT
	**H3122**	WT	WT	WT	WT	WT	Mut
	**H1792**	mut - G12C	WT	WT	WT	WT	WT
	**H1650**	WT	Mut	WT	Mut	Mut	WT
	**H2009**	mut - G12A	WT	WT	WT	WT	WT
***RICTOR* non-amplified**	**HCC193**	WT	WT	WT	WT	WT	WT
	**H1819**	WT	WT	WT	WT	WT	WT
	**A549**	mut - G12S	WT	Mut	WT	WT	WT
	**Calu6**	mut - Q61K	WT	WT	WT	WT	WT
	**HCC44**	mut - G12C	WT	Mut	WT	WT	WT
